# Supplementary material for: Effect of Dispersed ZrO2 Particles on Microstructure Evolution and Superconducting Properties of Nb-Ti Alloy
Source: Materials (Basel). 2024 Dec 4;17(23):5946. doi: 10.3390/ma17235946 (PMC11643743; doi:10.3390/ma17235946)
Supplement: Supplementary file 1 [file materials-17-05946-s001.zip › supplementary materials.pdf]

Supplementary materials: Effect of dispersed ZrO_2 particles on microsructure evolution and superconducting properties of Nb-Ti alloy

Rafał Idczak[1]*, Robert Konieczny[1], Wojciech Nowak[1,2], Wojciech Bartz[3], Michał Babij[2]

[1]Institute of Experimental Physics, University of Wrocław, pl. M. Borna 9, 50-204 Wrocław, Poland
[2]Institute of Low Temperature and Structure Research, Polish Academy of Sciences, ul. Okólna 2, 50-422 Wrocław, Poland
[3]Institute of Geological Sciences, University of Wrocław, ul. Cybulskiego 30, 50-205 Wrocław, Poland

S1 Additional information: scanning electron microscopy

Fig. S1 shows four randomly selected SEM/EDXS spectra prepared for 20 h (top panels) and 60 h (bottom panels) samples of Powders B. Table S1 shows the weight content of each element for the individual SEM spectra. The SEM measurement was performed under low vacuum conditions, so oxygen was not considered. Similarly, zirconium which was below or at the edge of the detection threshold.

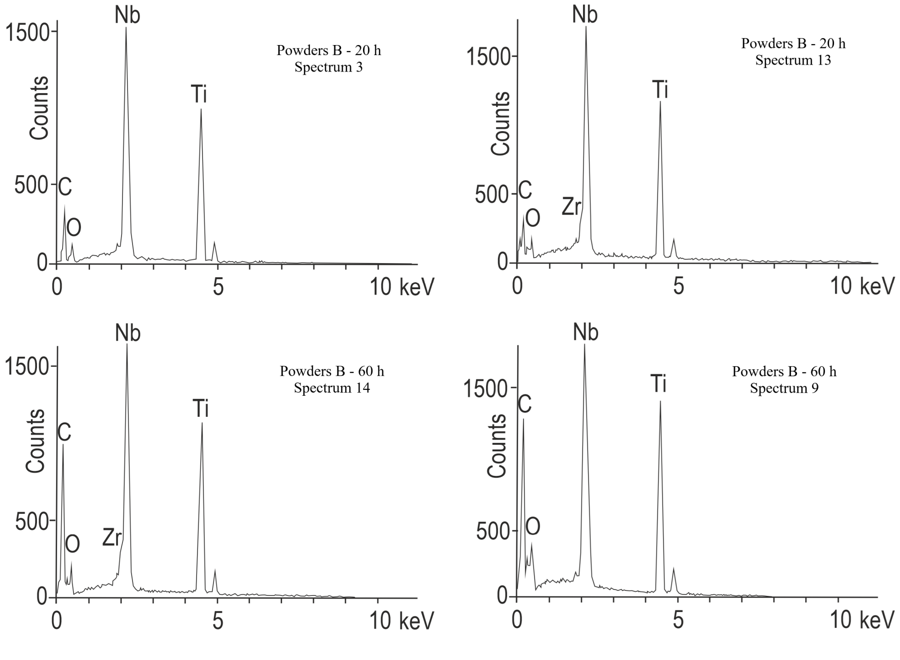

Figure S1. Randomly selected example SEM/EDXS spectra for Powders B 20 h and 60 h samples.

Table S1. The weight content of the SEM/EDXS spectra shown in Figure S1

Powder B	Spectrum	wt.% C	wt.% Ti	wt.% Nb	wt.% Zr
20 h	3	20.3(0.4)	37.0(0.7)	43.7(0.9)	0.0
	13	15.6(0.7)	36.3(0.8)	42.7(0.9)	5.5(2.8)
60 h	9	37.4(0.7)	30.8(0.6)	31.8(0.6)	0.0
	14	51.3(1.0)	20.7(0.4)	26.2(0.5)	1.8(0.9)

S2 Additional information: magnetic measurements

Figures S2 and S3 show, respectively, all the collected real χ' and imaginary χ'' dependencies of the ac-susceptibility component and the mass magnetic susceptibility as a function of temperature for Powders A. Analogous summaries for Powders B can be seen in Figures S4 and S5.

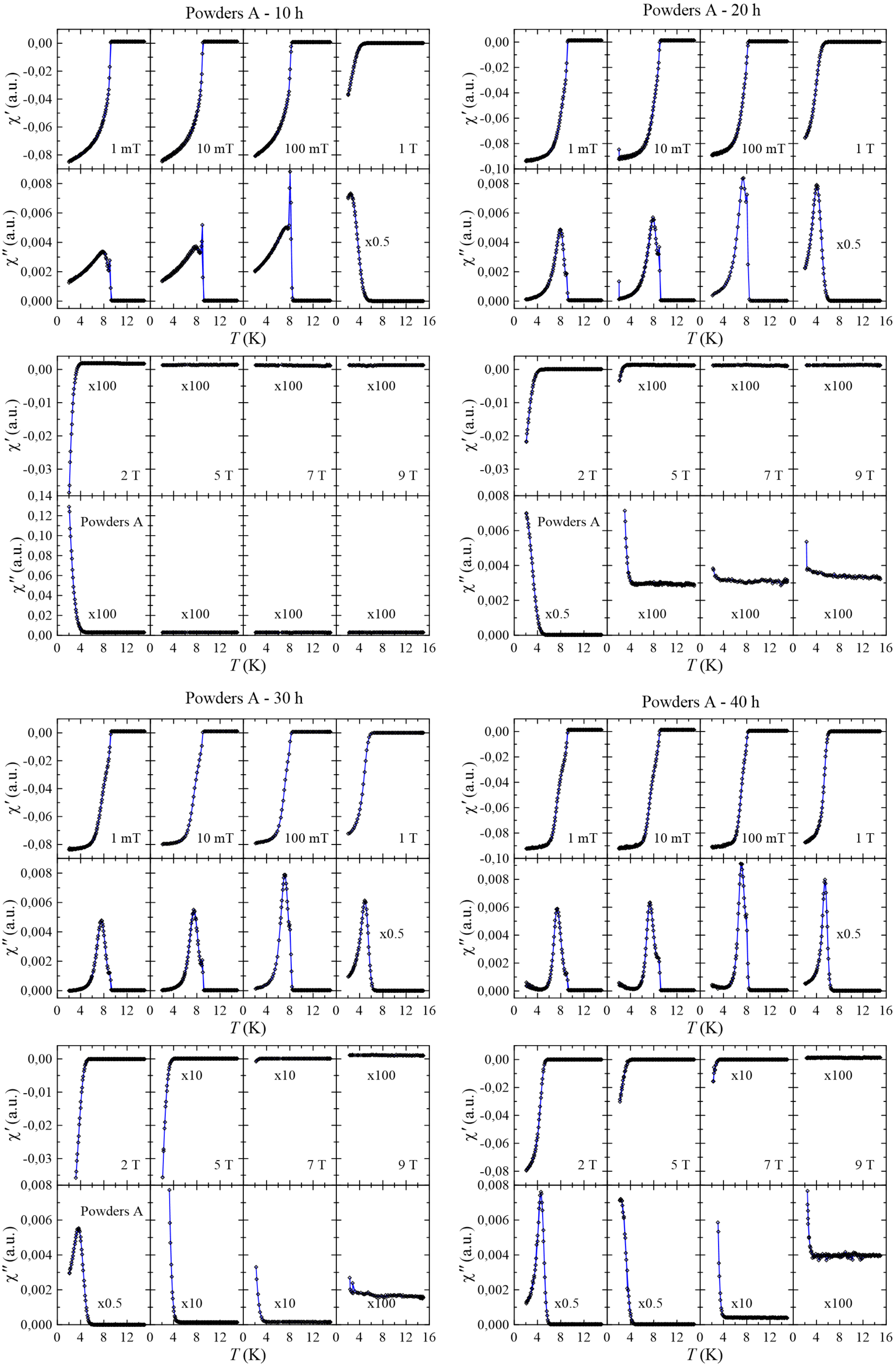

Figure S2. Real χ' and imaginary χ'' part of susceptibility of powders A measured as a function of temperature in several applied magnetic fields $\mu_0 H$. Solid lines were added to guide the eye.

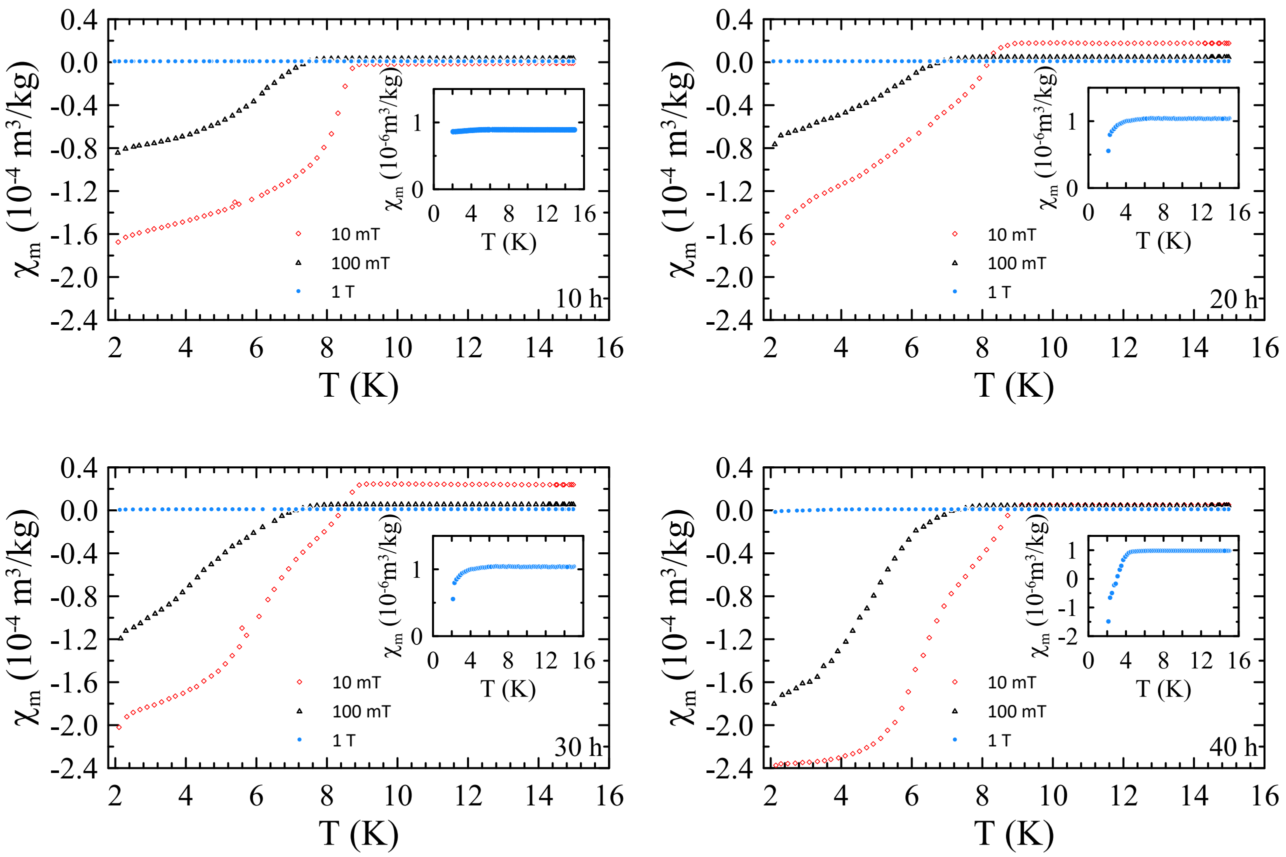

Figure S3. Mass magnetic susceptibility χ_m of Powder A samples measured as a function of temperature in a several applied magnetic fields in zero-field cooling (ZFC) regime. Solid lines were added to guide the eye.

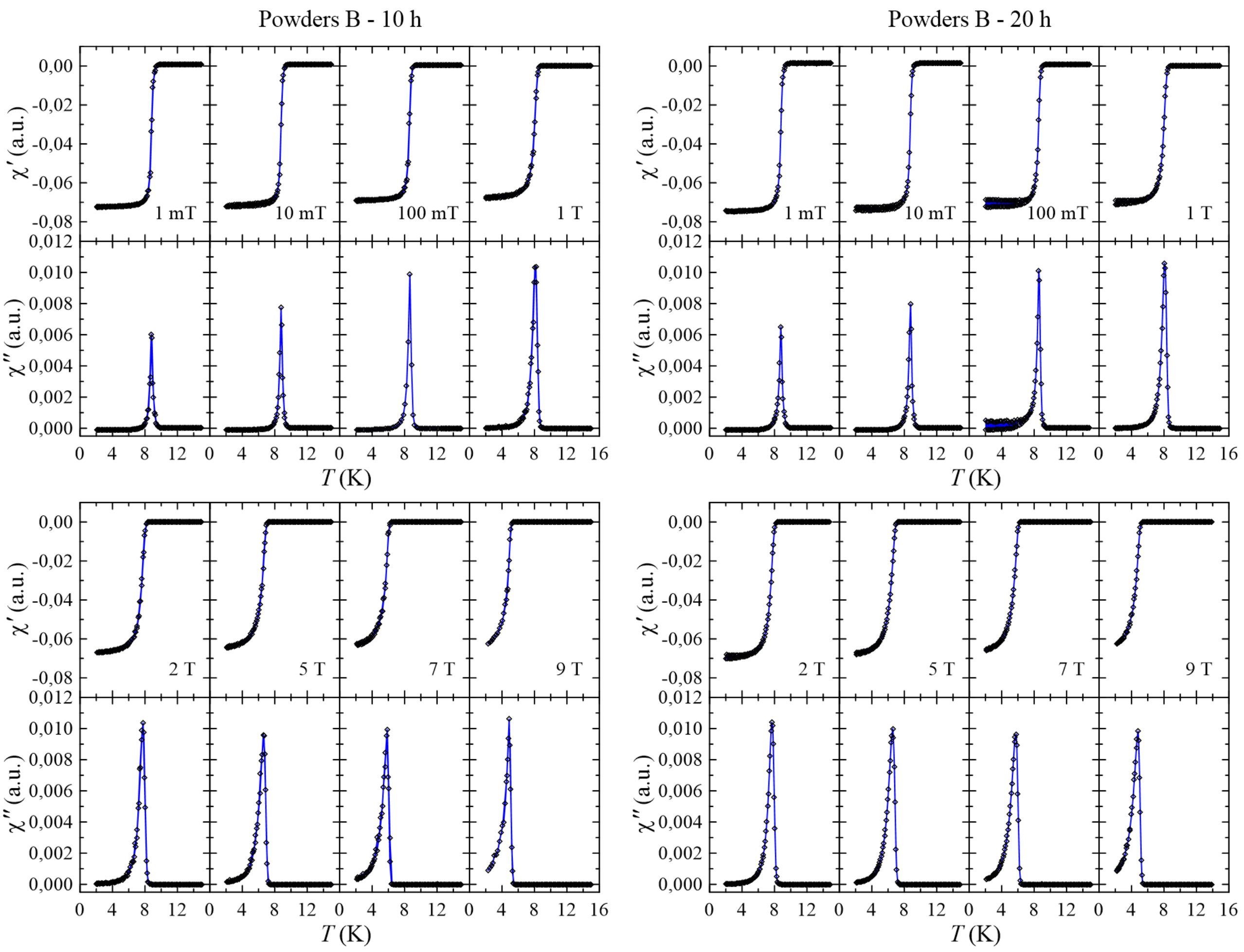

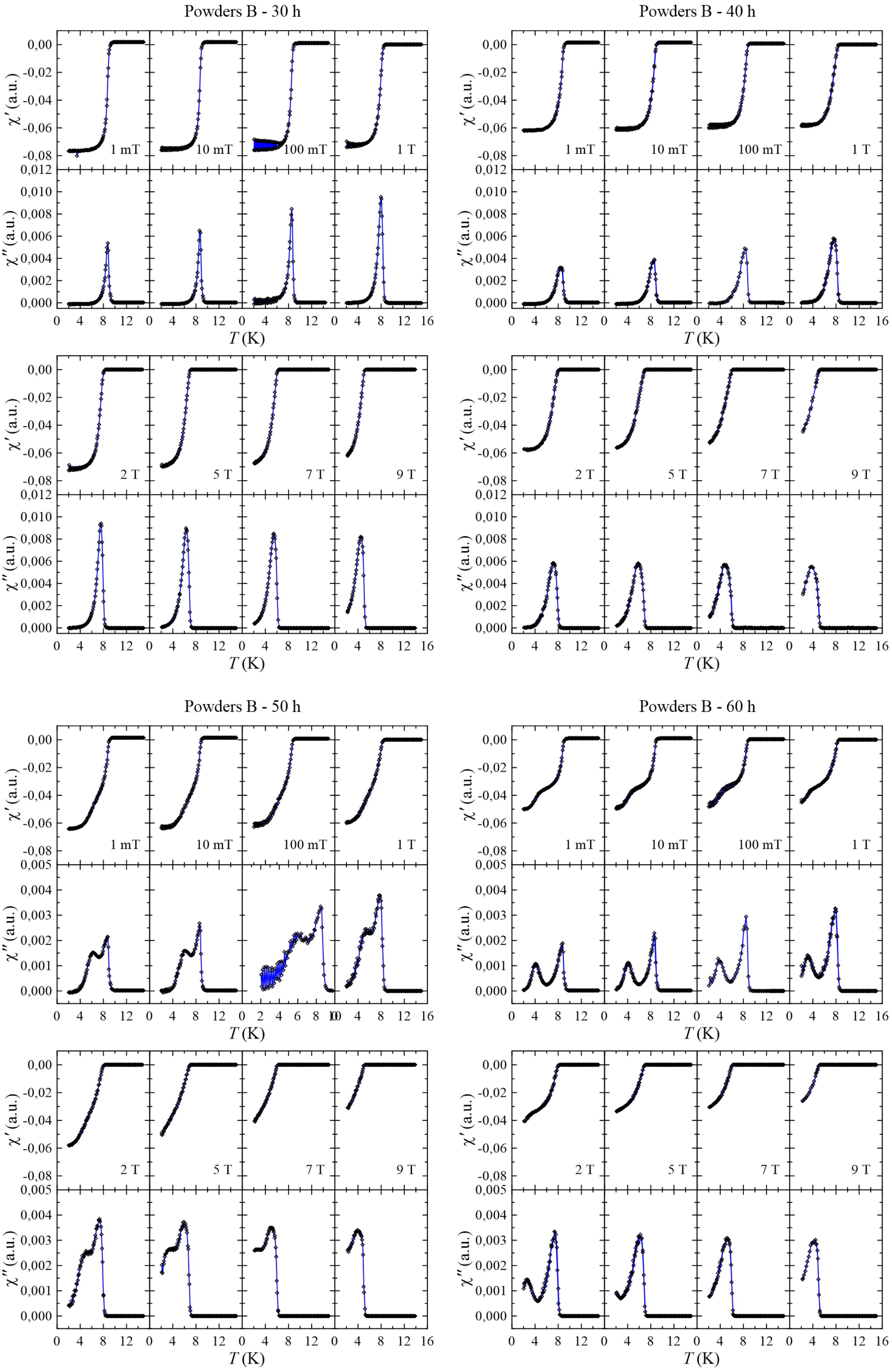

Figure S4. Real χ' and imaginary χ'' part of susceptibility of powders B as a function of temperature in several applied magnetic fields $\mu_0 H$. Solid lines were added to guide the eye.

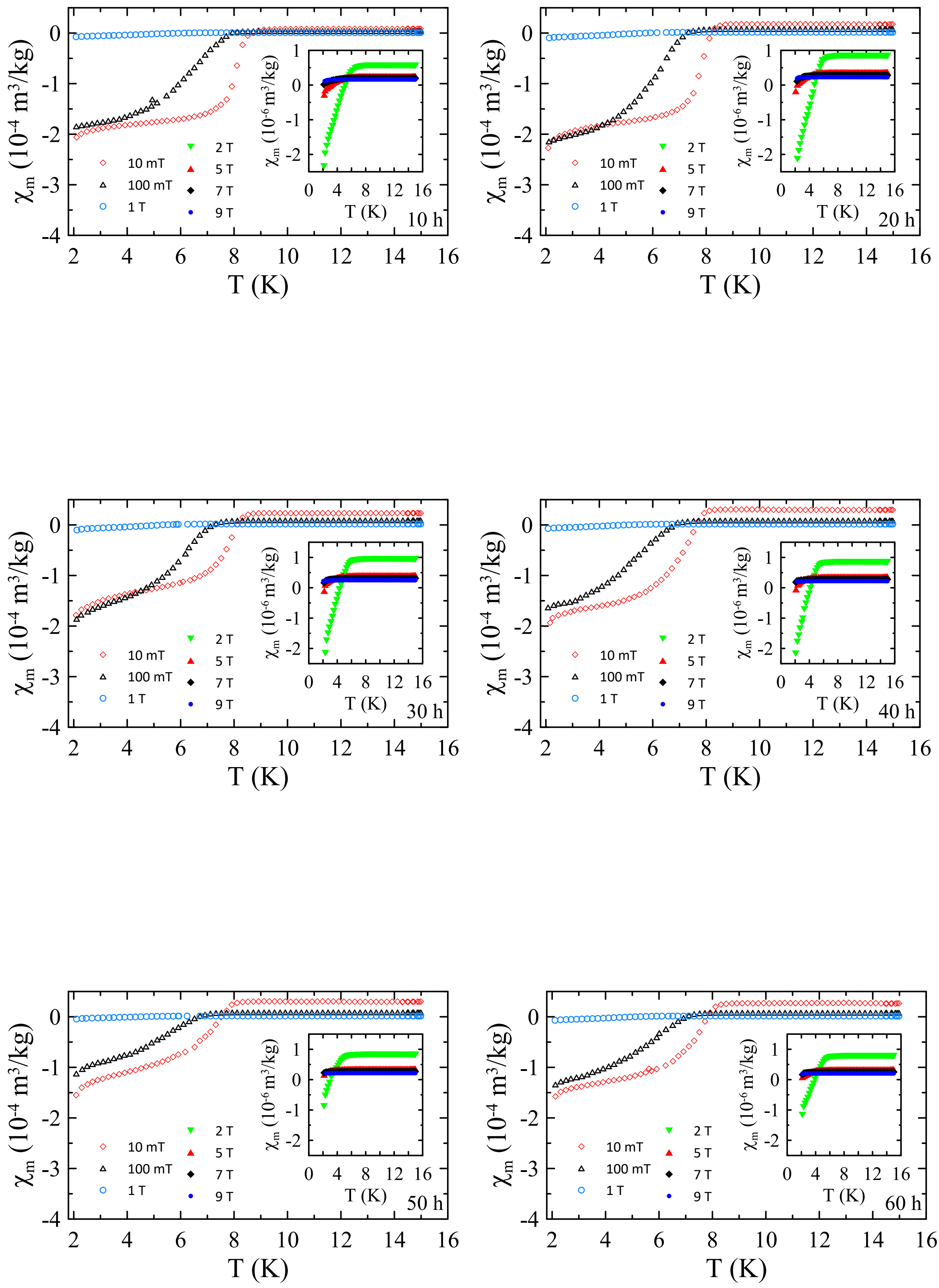

Figure S5. Mass magnetic susceptibility χ_m of Powder B samples measured as a function of temperature in a several applied magnetic fields in zero-field cooling (ZFC) regime. Solid lines were added to guide the eye.
